# Functions of Uninflatable in the *Drosophila melanogaster* wing and notum

**DOI:** 10.1371/journal.pone.0344871

**Published:** 2026-05-15

**Authors:** Taylor Zhang, Juliet E. Jones, Laura J. Yee, Sebastian C. Sy, Monika Karki, Manshi Patel, Seniha Elcik, Kathleen M. Beckingham

**Affiliations:** 1 University of Arkansas for Medical Sciences, Little Rock, Arkansas, United States of America; 2 Department of Biosciences, Rice University, Houston, Texas, United States of America; 3 Department of Obstetrics and Gynecology, Long School of Medicine, San Antonio, Texas, United States of America; 4 Department of Neuroscience and Experimental Therapeutics, Texas A&M University, College Station, Texas, United States of America; 5 UT Southwestern Medical School, Dallas, Texas, United States of America; Baylor College of Medicine, UNITED STATES OF AMERICA

## Abstract

The *Drosophila* gene *uninflatable* (*uif*) encodes a conserved insect protein, first identified for its roles in the development and endopolyploid growth of several larval tissues. Uif is a transmembrane protein and its large extracellular domain contains several protein-protein interaction motifs, including multiple EGF (**E**pidermal **G**rowth **F**actor-like) repeats. More recent studies have established that Uif can interact with Notch, a major regulator of *Drosophila* growth and differentiation, through its EGF repeats and have identified ways that Uif binding can modify Notch behavior. Endocytosis of Notch-Uif complexes into a particular class of endosomes has also been identified and implicated in cell fate decisions in the sense organs of the notum (thorax). We have examined *uif’s* functions in the *Drosophila* wing to assess possible roles in 1) growth of a mitotically derived tissue, 2) cell fate decisions in specialized wing structures, and 3) *Notch*-dependent processes. We used previously characterized Gal4 lines and RNAi constructs to suppress *uif* and *Notch* in different wing compartments. In addition to a role in mitotic growth throughout the wing, we have identified two new *uif* activities that are also shared with *Notch*: 1) regulation of pigment synthesis within the wing cuticle and 2) control of chemosensory sense organ number in the anterior wing margin through a role in an apoptosis-related mechanism. However, *uif* does not participate in two roles of *Notch* that regulate cell fate decisions: sense organ differentiation and formation of wing vein tissue. Given the similarities in the development of the notal and wing margin sense organs, we investigated further the previously proposed role for *uif* in differentiation of these structures on the notum. We found that loss of *uif* affects the growth of the bristles of the notal microchaete sense organs, but not their differentiation.

## Introduction

Multicellular organisms use a variety of growth mechanisms to create their individual tissues [1–6]. Defining the molecular bases of these differing pathways, and understanding how they relate to one another, has significance in terms of identifying evolutionary connections and providing insights into human health and pathology. Most tissues in *Drosophila* larvae grow by an unusual process termed endopolyploidy in which the nuclear and cytoplasmic division phases of metaphase are entirely eliminated and large cells with nuclei containing many copies of the genome are generated. We determined that the gene *uninflatable (uif*) has a role in the growth of several larval endopolyploid tissues including the tracheae [7]. However, *uif* is also expressed in the imaginal wing discs and in pupal wings during early cuticle formation [8], suggesting roles in mitotic growth and possibly differentiation of adult tissues.

Notch is a major player in *Drosophila* wing development, with roles in both growth and differentiation [9–14]. Uif is a transmembrane protein that has multiple protein-protein interaction motifs, including EGF repeats, in its extracellular domain [15]. Given that Notch interacts with its ligands Delta and Serrate through EGF repeats, prior studies have addressed the possibility that Uif may also be a Notch ligand. Xui et al. [16] investigated a possible interaction of Uif with Notch in wing development by overexpressing Uif, or a modified derivative of Uif, in the wing imaginal disc. Neomorphic phenotypes resulting from inhibition of Notch signaling components were produced together with evidence that these arose from an interaction between Notch and Uif on the same plasma membrane. However, despite this evidence for a Notch-Uif interaction, this group could not identify a wild type function for Uif in the wing using an RNAi-based approach.

Loubery et al. [5] also identified binding between Notch and Uif when co-expressed in cultured *Drosophila* cells and identified four EGF repeats in Uif that mediate this interaction. This study further linked an interaction between Notch and Uif to the well-established role of Notch in differentiation of the external sense organs (esos) on the adult thorax/notum [11,12]. These esos develop from a patterned array of sense organ precursor (SOP) cells. Each SOP undergoes two mitotic divisions to produce four cells that differentiate into the components of the eso. Notch signaling controls both the initial designation of SOP cells and the differentiation of the initial SOP mitotic progeny (cells termed pIIa and pIIb) and their subsequent descendants [12]. In an earlier study this group identified a route for preferentially directing Notch on a SOP cell plasma membrane into only one of its daughter cells (the pIIa cell) via a class of endosomes carrying the protein Sara [17]. In Loubery et al. [5] they provided evidence that Uif is required for this movement of Notch on Sara endosomes. In its absence, Notch transfer to pIIa failed, resulting in a low level (<10%) of cell fate transformations in the progeny of both pIIa and pIIb.

Based on this prior work and the current availability of functional *uif* RNAi’s [5,15] and *Notch* RNAi constructs [18,19], we have used Gal4 lines with known expression patterns in the wing to identify functions of *uif* in this organ and to compare its roles to those of *Notch*. We have also investigated *uif* and *Notch* function in the formation of eso’s on the notum for comparison to the findings of Loubery et al. [5]

## Materials and methods

### Fly stocks

**Sources for Gal4 lines used in this study.** *nub*-Gal4 - Bloomington Drosophila Stock Center (BDSC) #25754; *ptc*-Gal4 559.1 – BDSC #2017; C96-Gal4/*TM3 Sb Ser* – gift of Dr. Hugo Bellen; *cut*(ue)-Gal4 – BDSC #27327; *y w pnr*-Gal4/*TM3* {*pUAS-y.C}MC2 Ser* – BDSC #3039.

**Sources for UAS-RNAi lines used in this study.** *uif* RNAi‘s #V1050 and #V1051 – Vienna Drosophila Resource Center (VDRC) NOTE- #V1050 is no longer available from the VDRC; *Notch* RNAi’s #V1112 and #V100002– VDRC; *mgl* RNAi - BDSC #29324. **Other stocks.** UAS-p35 – gift of Dr. Hugo Bellen; *w N*^*5419*^*FRT19A/FM7c twi > GFP* – gift from Dr. Shinya Yamamoto; UAS-mCherry – gift of Dr. Venken Koen; UAS-nls-GFP – BDSC #4775. Canton-S wild type – gifts from Dr. Herman Dierick and Dr. Brigitte Dauwalder. Standard *Drosophila* food was used. Stocks were maintained at 18^0^C and 22^0^C.

### Wing and thorax imaging

Wing discs were prepared as described in Capodevila et al. [20]. Wings were dissected from anesthetized flies. Wings from males and females were collected separately in batches of ~10, transferred to clean microscope slides, and washed briefly with isopropanol. After drying they were mounted in Permount. No significant differences between male and female wings were detected and wings from both sexes were used for analysis. Thoraces were treated with 10% KOH for 10 minutes at 85^0^C to dissolve internal tissue, washed three times with 75% alcohol and mounted in 70% glycerol. Wings were imaged on the Zeiss M2 Axioimager microscope of the Shared Equipment Authority at Rice University. After dissection thoraces were imaged at Baylor College of Medicine using a Leica MZ16 stereomicroscope system designed to provide extended depth of field. Quantitation of various wing and notum features was performed using NIH image J.

## Results

**Fig 1:**
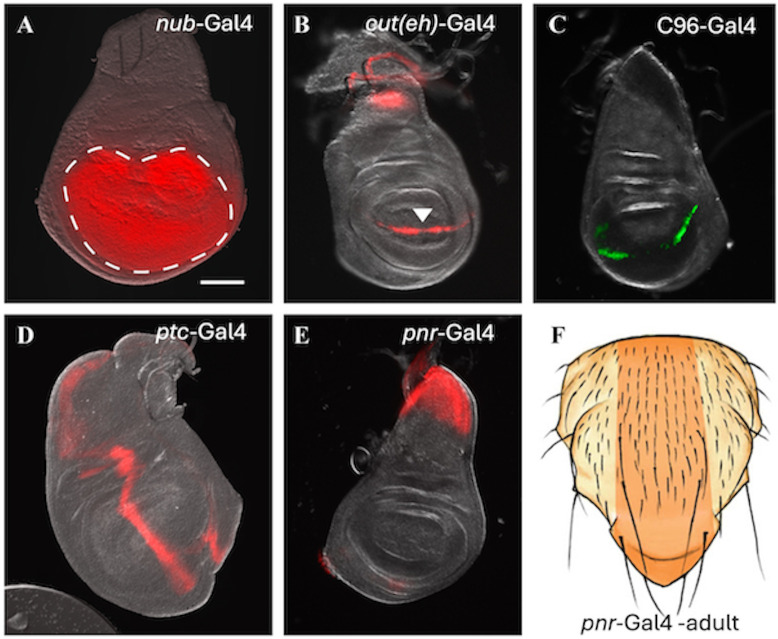
Wing disc expression patterns for Gal4 lines used in these studies. Expression patterns for wing discs from third instar or wandering larvae are shown. **A, B**, **D,** and **E** show Gal4 lines driving UAS-mCherry. For **C**. UAS-nuclear-GFP was used. **A.** *nubbin (nub)*-Gal4 is expressed throughout the prospective wing blade region (outlined with the dashed white line). **B.** The *cut*(ue)-Gal4 driver is expressed around the future wing margin where the prospective dorsal and ventral wing blade surfaces meet. This image shows the late expression of *cut*(ue)-Gal4 in wandering larvae. A noticeable decrease in expression occurs at the point where the dorsal-ventral and anterior-posterior boundaries meet at the prospective wing tip (white arrowhead). **C.** The C96-Gal4 driver is also expressed around the dorsal-ventral wing border and in late wandering larvae, loss of expression starts at the same point at the wing tip and spreads around the margin as seen with *cut*(ue)-Gal4. The wing tip is very sensitive to loss of *cut* activity [23] and the decline in *cut*(ue) and C96 activity at the wing tip as pupation approaches may contribute to that phenomenon. **D.** The stripe of *patched* (*ptc)*-Gal4 expression corresponds to the anterior/posterior boundary for pattern formation in the wing. **E**. In larval life, *pannier* (*pnr*)-Gal4 expresses in the notum (thorax) primordium. The region affected by *pnr-*Gal4 in the adult thorax is shown in **F.** as an orange stripe. Scale bar in **A.** = 50 μm.

The expression patterns of the five Gal4 lines used in this study are shown in Fig 1. Stocks containing two different *uif* RNAi’s [5,15] V1050 (here called *uif* RNAi-1) and V1051 (*uif* RNAi-2) and two different *Notch* RNAi’s [18,19] V1112N (*Notch* or *N* RNAi-1) and V10002N (*Notch or N* RNAi-2) were used in our experiments. Each pair of RNAi’s produced very similar phenotypes, as exemplified by the comparisons shown in S1 Fig. We conclude that each of these RNAi’s produces specific knockdown of the targeted gene. Some crosses were performed at both 25^0^C and 29^0^C to assess maximal phenotypic effects. The quantitative data for all of the experiments described here are presented in the S1-S11 Tables of the Supporting Information.

At the end of development, the only live cells in the wing are found in its neural structures [21]. The wing blade is formed by the apposition of the dorsal and ventral cuticles, which are secreted by the blade epithelial cells. Immediately after emergence from the pupal case (eclosion) these cells undergo apoptosis, their corpses are swept into the thorax, and the final blade tissue is composed of the dorsal and ventral cuticles separated by a narrow, cell-free, lumen [22]. However, the footprints of the secretory epithelial cells are embedded in the cuticle, along with the short hairs (trichomes) produced by the epithelial cells. When describing “cells” in final wing structures, we are referring to these cell footprints.

### Like *Notch*, *uif* is required for growth in the wing

**Fig 2:**
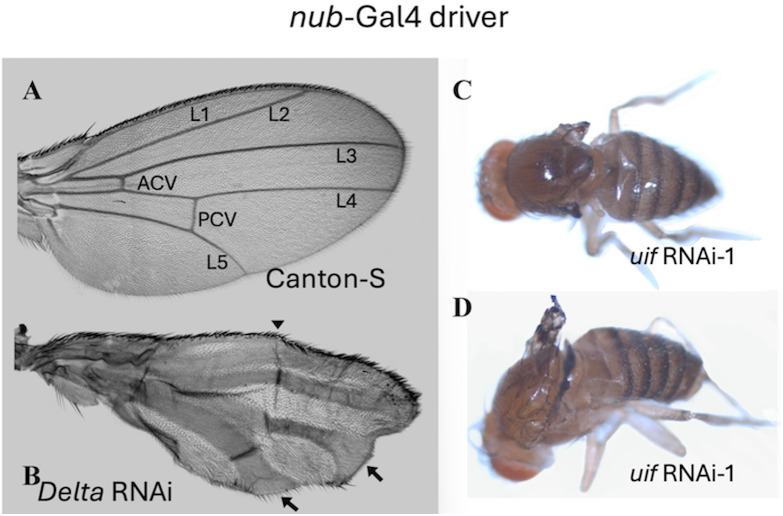
*nub*-Gal4 knockdown suggests a role for *uif* in wing growth as opposed to differentiation. **A.** *nub*-Gal4 > Canton-S control wing showing wing vein nomenclature. **B.** *nub*-Gal4 > *Delta* RNAi wing. *Delta* exemplifies a Notch ligand with major roles in cell differentiation. Arrows indicate greater vein expansion at the margin. Arrowhead marks fusion of the L1 and L2 veins. **C, D.** Two extremes of *nub*-Gal4 > *uif* RNAi-1 phenotype. Almost no wing tissue is present on fly **C**, and on fly **D.** some crumpled, pigmented tissue is everted.

We used the *nubbin(nub)*-Gal4 [24] driver, which expresses throughout the wing blade (Fig 1A), to assess global knockdown of *uif* and *Notch* in blade epithelial cells. A control wing blade is shown in Fig 2A. We also examined the wings of *nub*-Gal4 > *Delta* RNAi flies (Fig 2B) to allow comparison of any *uif* phenotypes detected to those of a well-characterized Notch ligand that functions by EGF-mediated binding. *nub*-Gal4 > *uif* RNAi-1 wings showed extreme defects in growth and wing expansion. About half of the adults examined had small wing stumps on the thorax instead of wings (Fig 2C). The remainder had short, blackened wings that were too crumpled to analyze (Fig 2D). Almost 100% of *nub*-Gal4 > *Notch* RNAi-1 flies died as pupae with very short, folded blackened wings, but surprisingly, a very small number (five) eclosed after a developmental delay of at least seven days. These flies also had tiny, blackened, wings. In contrast, the *nub*-Gal4 > *Delta* RNAi wings were almost normal size with one major development defect: the wing veins were massively expanded in width (Fig 2B). This finding reflects the inhibitory role of *Delta* on *Notch* specification of vein cells [25]. *Delta* knockdown was strongest where the veins contact the wing margins. Although Delta and Uif both use an EGF repeat-based mechanism to interact with Notch, a role in wing growth, as opposed to wing differentiation, appears likely for Uif. Quantitative data, S1 Table.

### Both *Notch* and *uif* support growth in the cells of the wing blade

**Fig 3:**
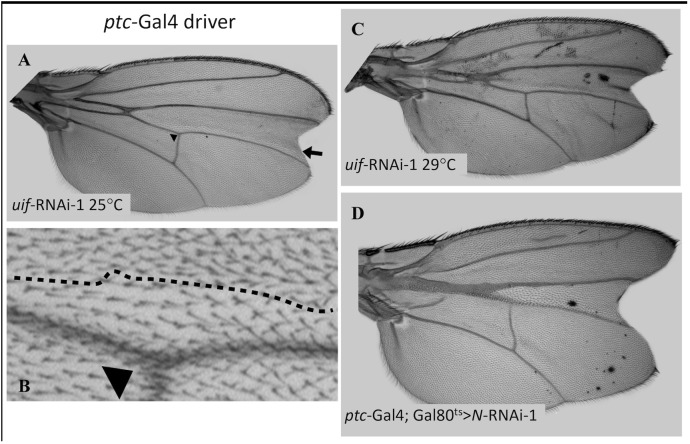
*uif* regulates growth in wing blade cells. **A.** *ptc*-Gal4 > *uif* RNAi-1 at 25^0^C. Arrow indicates the position of the border between the stripe of *ptc*-Gal4 > *uif* RNAi-1 cells and the stripe of unaffected cells. Arrowheads in **A.** and **B.** provide a reference point for enlarged tissue in **B. B.** Higher magnification image of the border between the *ptc*-Gal4 > *uif* RNAi stripe and control tissue in the wing shown in **A. C.** *ptc*-Gal4 > *uif* RNAi-1 wing at 29^0^C. Note increased pigmentation. **D.** *ptc*-Gal4; *Gal80*^*ts*^>*Notch* RNAi at 29^0^C. Note terminal wing scallop as seen in wing **C**.

For an investigation of *uif* knockdown specifically on the epithelial cells of the wing blade, we used *ptc*-Gal4 [26] to drive *uif* RNAi in a proximal-to-distal stripe of epithelial cells between the L3 and L4 wing veins (Fig 1D). (See Fig 2A for wing vein nomenclature). This longitudinal *ptc*-Gal4 stripe does not occupy the entire horizontal width of the tissue between L3 and L4 and as a result, a stripe of wild type cells, abutting vein L4, runs next to the stripe of *ptc*-Gal4 expression (see Fig 3A, B). This arrangement made the effect of *uif* knockdown on cells in the *ptc*-Gal4 stripe easily identifiable: they were noticeably smaller and more pigmented than their wild type neighbors (Fig 3A, B). We established that there were 50% more small cells in a defined area of the *ptc*-Gal4 > *uif* RNAi-1 stripe than in a comparable wild type control region (S2 Table). A compensatory mechanism must provide enough of the smaller *uif* knockdown cells to occupy the space designated for the normal-size cells. The *ptc* expression stripe passes through the Anterior Cross Vein (ACV) and in *ptc-*Gal4 > *uif* RNAi-1 wings, this vein is essentially absent and replaced by a fusion of L3 and L4 (Fig 3A). Quantitative data, S3 Table.

The most striking feature of *ptc*-Gal4-*uif* RNAi wings is a large scallop/notch, spanning the wing margin at the distal end of the *ptc* stripe (Fig 3A, C). Scalloping (see below), particularly at the wing tip, is a defining characteristic of loss of the *Notch* growth function [14]. *ptc*-Gal4 > *Notch* RNAi flies did not survive to adulthood, so we used the temperature sensitive *Gal80*^*ts*^ allele to block *ptc*-Gal4 expression during larval life, followed by *Gal80*^*ts*^ inactivation at 29^0^C near the onset of pupation. Using this approach, a small number of *ptc*-Gal4; *Gal80*^*ts*^>*Notch* RNAi wings with striking similarities to the *ptc*-Gal4 > *uif* RNAi phenotype were isolated (Fig 3D). A scallop between L3 and L4, almost identical to the *uif* knockdown scallop, was present and a stretch of L3-L4 vein fusion replaced the ACV. However, the orderly suppression of cell growth in the *ptc* stripe of *ptc*-Gal4 > *uif* RNAi wings was not present. Instead, *Notch* control of growth was disorganized over long ranges, with some cells being larger than normal and producing an overall enlargement of the wing.

At 25^0^C, in addition to the increased pigmentation in the stripe of *ptc*-Gal4 > *uif* RNAi-1 cells described above, a small fraction of the wings also showed excess pigment deposition. At 29^0^C, **all** *ptc*-Gal4 > *uif* RNAi wings had regions with excess pigment (Fig 3C). The *ptc*-Gal4 > *Notch* RNAi-1 wings showed a milder version of this phenomenon (Fig 3D). Strikingly, this overproduction of pigment was not confined to the stripe of *ptc*-Gal4 expression but was randomly distributed throughout the wings. We refer to this phenomenon as ectopic pigmentation. Pigmentation problems were never seen for control wings at 25^0^C or 29^0^C. Quantitative data, S3 Table.

### Both *Notch* and *uif play* roles in the growth of the wing margin

We used two Gal4 lines (C96-Gal4 and *cut*(ue)-Gal4) to address the effects of *Notch* and *uif* knockdown on wing margin growth and differentiation of the margin’s external sense organs (esos). C96-Gal4 is expressed around the future wing margin during the third larval instar in a broad stripe that includes the two margin rows of cells that express *wingless (wg)* and four-five rows that express *cut* [27]. Both *wg* and *cut* are required for wing growth, *wg* throughout the wing and *cut* mainly at the margin, with both genes acting downstream of *Notch* [28,29]. The *cut*(ue)-Gal4 construct contains a *cut* enhancer initially thought to be expressed in the precursors to the wing margin mechanosensory esos [23], but a later study has provided convincing evidence that it expresses in the rows of margin *cut*^*+*^ cells (see above) that ultimately create the margin proper [30]. During third larval instar C96-Gal4 and *cut*(ue)-Gal4 activity extend around the entire developing wing margin. The later loss of their expression at the presumptive wing tip (Fig 1B, C) may explain the greater sensitivity of the wing tip to loss of *Notch* function during development [23].

The wing margin consists of three distinct regions: a pigmented anterior section with three rows of esos that ends at the distal tip of L2; a mildly pigmented stretch which runs from the L2 vein distal tip to the distal tip of vein L4 and contains two rows of esos and a final section with no obvious separate structure and two rows of non-sensory bristles. The phenotype induced by loss of *Notch* function that starts at the wing tip is termed scalloping or notching. The scallops are regions of lost margin tissue produced by failed growth, tissue death, and subsequently apoptosis to remove cellular remnants [31]. Scallops do not contain esos, reflecting the loss of the support functions provided by *cut*^*+*^ cells in the margin [30]. However scalloped regions are flanked by tufts of closely packed bristles. As an approach to assessing the contribution of *Notch* and *uif* to margin growth we looked for tissue loss and “scallop and tuft” formation in *Notch* and *uif* knockdown wings.

**Fig 4:**
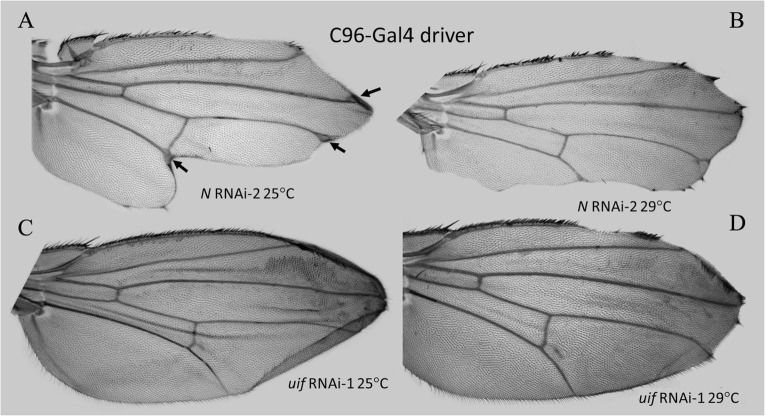
Comparison of *uif* and *Notch* loss of function at the wing margin using the C96-Gal4 driver. **A.** C96-Gal4 > *Notch* RNAi-2 at 25^0^C. Arrows indicate wing vein expansion at wing margin. Note extensive posterior wing tissue loss and anterior margin loss. **B.** Major blade loss but clear scalloping and tufting on one wing of C96-Gal4 > *Notch* RNAi-2 at 29^0^C. **C.** C96-Gal4 > *uif* RNAi-1 at 25^0^C. Note “cupping” (see text) at wing tip. **D.** C96-Gal4 > *uif* RNAi-1 at 29^0^C. All wings showed wing tip scalloping at 29^0^C which allowed these wing tips to flatten. Note ectopic pigmentation of C96-Gal4 > *uif* RNAi-1 wings in C. and **D.**

At 25^0^C, C96-Gal4 > *Notch* knockdown by either *Notch* RNAi produced a small number of wings with archetypical “scallop and tuft” margins, but the major phenotype was extensive loss of wing tissue, deep into the blade, that eliminated virtually all of the posterior margin bristles and at least half of the anterior margin (Fig 4A). The wing veins were expanded at the wing tip (Fig 4A, arrows) reflecting, in this case, loss of a feedback regulatory role of *Notch* in vein formation [25]. At 29^0^C this tissue loss was even more extensive and only a single wing from the two C96-Gal4 > *Notch* RNAi’s genotypes had a “scallop and tuft” margin (Fig 4B). Quantitative data, S4 Table.

**Fig 5:**
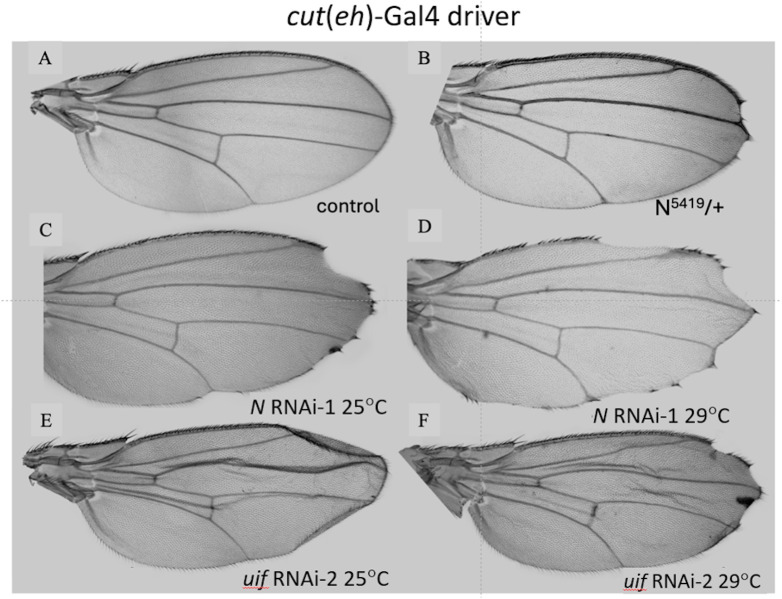
Comparison of *uif* and *Notch* loss of function at the wing margin using the *cut*(ue)-Gal4 driver. **A.** Control wing. **B.** Reference example of ”scallop and tuft”phenotype on the tip of a *N* ^*5419*^ heterozygous dominant mutant wing. **C.** *cut*(ue)-Gal4 > *Notch* RNAi-1 at 25^0^C. **D.** *cut*(ue)-Gal4 > *Notch* RNAi-1 at 29^0^C. Enhanced scalloping is seen at 29^0^C. **E.** *cut*(ue)-Gal4 > *uif* RNAi-2 at 25^0^C. Note mild “cupping” at wing tip. **F**. *cut*(ue)-Gal4 > *uif* RNAi at 29^0^C. Scalloping only detected at 29^0^C.

At 25^0^C, the *C96-*Gal4 > *uif* RNAi knockdown phenotype was distinctly different from that of *Notch*. The wings showed very little tissue loss in the margin but had a “cup-like” structure at the wing tip, created by upward bending of the wing edge around a pointed tip (Fig 4C). This “cupping” phenotype involved smaller wing blade tip cells outside the margin and presumably strong growth inhibition in the margin tip due to local loss of *cut* and *wg* function. These wings also had longitudinal creases presumably caused by mechanical compression induced by the cupped tip. At 29^0^C, the “cupped” phenotype was completely absent, and all wings had a “scallop and tuft” phenotype (Fig 4D) similar to that of the dominant *Notch* mutation *N*^*5419*^ (Fig 5B). These wings were flat suggesting that the scalloping released the mechanical tension present in the “cupped” wings. Quantitative data, S4 Table.

All wings from the two *cut*(ue)-Gal4 > *Notch* RNAi crosses showed no loss of internal blade tissue (Fig 5C, D) confirming the limitation of the *cut*(ue)-Gal4 driver expression to the margin. At both 25^0^C and 29^0^C all of these *Notch* RNAi wings showed the “scallop and tuft” margin phenotype. At 25^0^C, scallops were mainly at the wing tip (Fig 5C) whereas at 29^0^C more proximal scallops were also present (Fig 5D). At 25^0^C both of the *cut*-Gal4 > *uif* RNAi crosses produced a weaker form of the “cupping” phenotype (Fig 5E) seen with C96-Gal4 > *uif* RNAi wings (see above) but at 29^0^C, wings had a “scallop and tuft” phenotype very similar to the *cut*(ue)Gal4 > *N* RNAi phenotype at 25^0^C (Figs 5C, F). Given that gene knockdown is more effective at 29^0^C than at 25^0^C, we conclude that the three phenotypes seen amongst these wings represent mild (“cupping”), moderate (“scallop and tuft”) and severe (major tissue loss) versions of loss of *Notch’s* growth function in the wing margin and reveal a minor role for *uif* in this activity. Quantitative data, S5 Table.

Most of the wings from C96-Gal4 > *uif* RNAi crosses produced ectopic pigmentation in the blade (Fig 4C, D) but the C96-Gal>*Notch* RNAi wings and all of the *cut*(ue)-Gal4 wings showed very little of this phenomenon.

### *uif* knockdown does not phenocopy the effects of *Notch* knockdown on cell fate decisions in the margin

Development of the wing margin esos is related to that of the microchaetes and macrochaetes on the thorax (notum) [11,32,33]. As on the notum, *Notch* regulates the development of the founder SOPs that then produce sister pIIa and pIIb cells. Subsequently pIIa divides asymmetrically to generate the bristle and socket cells of the eso. Failed pIIa asymmetric division yields esos with two shafts or isolated large sockets. Loubery et al. [5] have provided evidence that *uif* is required in the notal microchaete esos for this asymmetrical division of pIIa. On suppression of *uif* they found evidence that a small number of pIIa cells divided symmetrically to yield isolated sockets. To address the possibility of a similar *uif* role in the wing margin, we examined a subset of anterior margin esos on the *uif* and *Notch* knockdown wings described above. Three rows of esos are embedded in the anterior wing margin [11,12]. The dorsal anterior margin has a row of stout mechanosensory eso bristles at the wing edge and an internal row of more widely spaced chemosensory esos with slender bristles. We focused on the bristles and sockets of the esos in these two rows.

**Fig 6:**
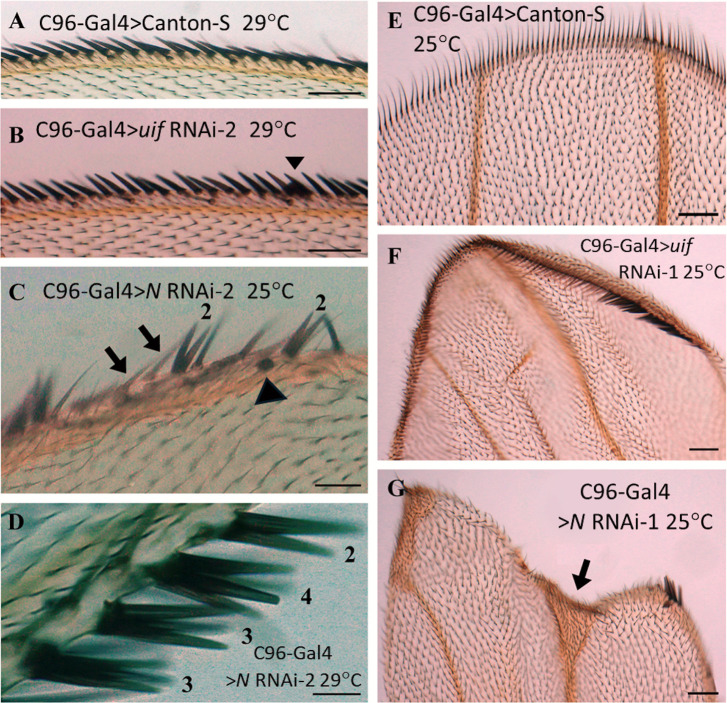
Cell fate changes in the margin produced by *Notch* knockdown but not *uif* knockdown. **A**. Control anterior wing margin at 29^0^C. **B.** C96-Gal4 > *uif* RNAi-2 wing margin at 29^0^C. The eso bristles were unaffected by loss of *uif* activity, although pigment accumulated between bristles of some wings (arrowhead in **B**). **C, D.** C96-Gal4 > *Notch* RNAi-2 wings. **C.** *Notch* knockdown produced double bristle shafts (labeled 2), isolated sockets (arrowhead in **C.**), pale stout bristles lacking in pigment (arrows). Loss of *Notch* also produced enlarged structures made up of three or four stout bristle shafts (labeled as 3 or 4 in **D.**). **E.-G. Crosses at 25**^**0**^**C**. Wing tips at the termini of veins L3 and L4 are shown. **E.** C96-Gal4 > Canton-S control, **F.** C96-Gal4 > *uif* RNAi-1, and **G**. C96-Gal4 > *Notch* RNAi wings. The wing tips from *Notch* RNAi crosses showed a change in cell differentiation at the margin (expanded wing tissue see arrow) whereas the tips of C96-Gal4 > *uif* RNAi wings were cupped, with pigmented hairs around the tip. Scale bars = 500 μm.

Knockdown of *uif* with C96-Gal4 provided no convincing effects on eso differentiation or the general arrangement of these esos within the margin (Fig 6A, B). Forty-eight wings (28 from 25^0^C crosses and 20 from 29^0^C) were examined and all had wild type arrays of stout and chemosensory bristles (Fig 6A, B). Two aberrant double stout bristle structures were detected amongst the 20 wings generated at 29^0^C (see S6 Table). However an unusual behavior of cells in the stout bristle lineage may explain the generation of these two structures rather than a defect in pIIa cell division (see Discussion). Wings from the *cut*(ue)-Gal4 > *uif* RNAi crosses produced no detectable structural changes to the esos. This lack of effect is consistent with the evidence that *cut*(ue)-Gal4 is not expressed in the margin esos [30]. Quantitation data, S6 Table)

Knockdown of *Notch* with C96-Gal4 eliminated margin tissue around most of the wing edge (see Fig 4A, B) but stretches of the proximal anterior wing margin were present on most of wings (Fig 4A, B) and they were used to examine *Notch*-related effects on eso development. These margin fragments were disorganized with narrow regions missing many bristles (Fig 6C). But they contained striking examples of Notch-regulated cell fate transformations within the pIIa lineages of the stout mechanoreceptor esos. Weaker effects were detected for the chemosensory esos. At 25^0^C, every C96-Gal4 > *Notch* RNAi wing examined had on average four double-shafted stout bristle structures in these limited sections of margin (Fig 6C, D). Double bristles for the chemosensory esos were rarer. For both classes of esos, only a few isolated bristle sockets, on average 1–2 per wing, were detected (Fig 6C).

In addition to these expected *Notch regulated* transformations, at 29^0^C, multiple examples of unusual structures that, to our knowledge, have not been reported previously, were also present in C96-Gal4 > *Notch* RNAi wings. These were over-sized stout bristle structures formed with three or four stout shafts (Fig 6D). Mechanisms for the formation of these structures are addressed in the Discussion. A general role for *Notch* in the growth of the eso bristles, in addition to its role in their differentiation, was also revealed by our analysis. In regions where *Notch* knockdown produced a very narrow wing margin, esos were present but they had fewer, shorter, thinner, disorganized bristles that were, nevertheless, recognizable as either stout mechanosensor or slender chemosensor bristles (Fig 6C). This role for *Notch* could involve both direct action of *Notch* and indirect action of *Notch* via sustaining the wing margin tissue.

The widening of the wing vein termini at the margin of C96-Gal4 > *Notch* RNAi wings described earlier (Figs 4A and 6G) is a second example of a change in cell fate induced by loss of *Notch* activity. This phenotype was not seen with *uif* knockdown (Fig 6F). In contrast, the “cupped” tips of C96-Gal4 > *uif* RNAi wings lacked this cell fate change and had enhanced pigment deposition in the bristles spanning the wing tip (compare Fig 6E, F). Quantitative data, S6 Table.

### *Notch* and *uif* knockdown both affect pigmentation in the margin

**Fig 7:**
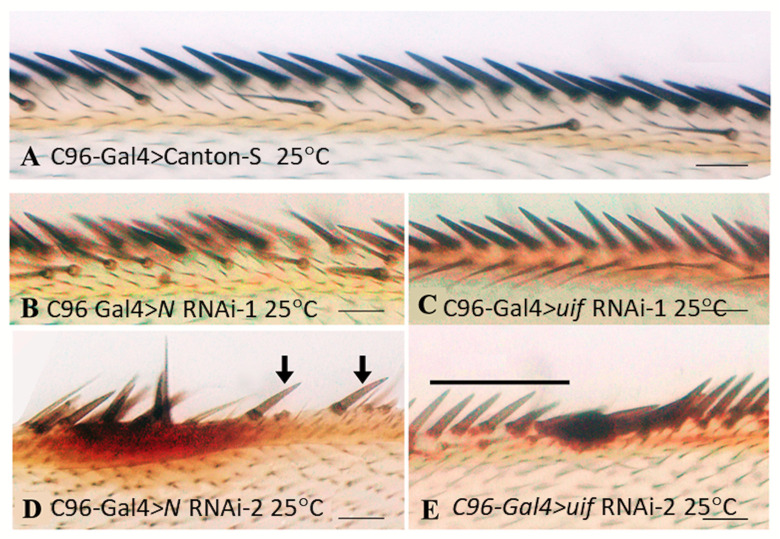
Shared margin phenotypes produced by loss of *Notch* and *uif* activity. Anterior wing margins from crosses at 25^0^C. **A.** C96-Gal4 > Canton-S control. **B.** C96-Gal4 > *Notch* RNAi-1, **C.** C96-Gal4 > *uif* RNAi-1, **D**. Gal4 > *Notch* RNAi-1. **E.** C96-Gal4 > *uif* RNAi-2. In control wings (**A.**) the chemosensory bristles (inner row) are positioned at approximately every fourth stout bristle. **B, C**. *uif* and *Notch* RNAi knockdown both generate supernumerary chemosensory organs in the inner row. Their arrangement in *Notch* RNAi margins is more disorganized because of other disruptions to the margin. **D.**, **E**. Both *uif* and *Notch* RNAi knockdown produce extracellular pigment in the wing margin often near stout mechano-sensory bristles without pigmentation (arrows in **D.** and black line in **E**). Scale bars = 500 μm.

In addition to ectopic pigment deposition in the wing blade, the C96-Gal4 driver also generated modest pigment anomalies in the margins for both *uif and Notch* knockdown. Extracellular pigment deposits accumulated at the base of the stout eso bristles (Fig 6B and Fig 7D, E). Some of these “blobs” were adjacent to abnormally pale stout eso bristles that had failed to accumulate pigment (Fig 7D, E), suggesting problems with both pigment uptake and pigment overproduction. Only one example of this phenomenon was seen with the *cut*(ue)-Gal4 driver, Quantitative data, S7 Table.

### Loss of *Notch* and *uif* function both induce overproduction of chemosensory esos in the anterior dorsal wing margin

The C96-Gal4 and *cut*(ue)-Gal4 crosses revealed a second shared phenotype for *Notch* and *uif* knockdown that affects the number of chemosensory esos of the inner anterior dorsal margin row. These esos are normally widely spaced and positioned in front of approximately every fourth stout bristle in the outer row (Fig 7A). With the C96-Gal4 driver, both *Notch* and *uif* RNAi’s produced additional chemosensory esos in the inner row (Fig 7B, C). For *Notch RNAi,* the absence of margin in stretches of C96-Gal4 knockdown wings (Fig 4A, B, and Fig 6C, D) limited the detection of additional esos, and only runs with 3–9 esos in poorly organized arrays were identified. In C96 -Gal4 > *uif* knockdown these supernumerary esos were in tightly organized rows, with longer arrays (12–31 esos) (Fig 7C) and an overall more penetrant phenotype. Quantitative data, S7 Table

*Knockdown with the cut*(ue)-Gal4 produced stronger effects. Without the margin disruptions present with the C96-Gal4 driver, *cut*(ue)-Gal4 > *Notch* knockdown produced esos arrays with up to 19 esos. The phenotype was strongest for *cut*(ue)>*uif* RNAi-2 which produced wings with runs of 30–54 esos. Quantitative data, S7 Table.

### Further analysis of the phenotypes identified for *Notch* and *uif*

1. **A pigmentation phenotype of *uif* and *Notch* mimics that of *Megalin***

**Fig 8:**
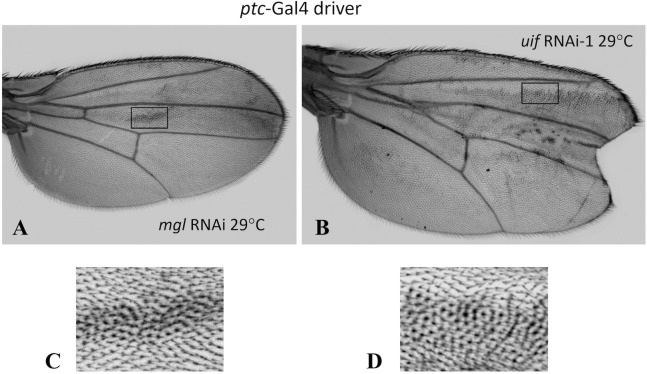
*uif* knockdown produces a similar but stronger effect on wing pigmentation than *mgl* knockdown. Whole wing images for **A.** *ptc*-Gal4 > *mgl* RNAi and **B.** *ptc*-Gal4 > *uif* RNAi-1 wings from crosses at 29^0^C. **C**. A patch of pigment puddles from the *mgl* RNAi wing shown in **A.** (see box on wing). **D.** Equivalent image for the *uif* RNAi wing in **B.** (see box on wing). Larger deposits of pigment are present in *ptc*-Gal4 > *uif* RNAi wings.

*uif* and *Notch* knockdown with the *ptc*-Gal4 and C96-Gal4 drivers produced ectopic wing blade pigmentation. Each driver produced the same three types of pigment aberration – i) cells with a mild, uniform increase in pigmentation (Fig 3A, B); ii) isolated, blobs of pigment (Fig 3C, D); and iii) puddles of pigment around the bases of the wing blade trichomes (Fig 8C, D). A trichome pigment puddle phenomenon has been reported for mutants of *megalin* (*mgl*), a Drosophila homolog of mammalian LRP2 [34,35]. Like Uif, Mgl has a long extracellular domain with EGF repeats. Wing pigment is generated extracellularly in the wing cuticle using components secreted by the blade epithelial cells. To initiate pigment production, the Yellow protein is secreted into the cuticle, but later, pigment formation is terminated via Mgl-mediated endocytosis of Yellow back into the cells [34]. When *mgl* is inactive, pigment production continues, leading to the puddling phenotype. We used the only available *mgl* RNAi [36] to compare *mgl* knockdown (Fig 8A) to *uif* knockdown (Fig 8B) using the *ptc*-Gal4 driver. Loss of function for both genes produced the same trichome puddles (Fig 8C, D) with *uif* knockdown producing a greater effect than *mgl* knockdown. As seen for loss of *uif* function, the pigment defects produced in *mgl* knockdown were widespread and not limited to the *ptc*-Gal4 stripe (Fig 8A). Although *mgl* and *uif* both contribute to pigment formation, *ptc-Gal4 > mgl* RNAi wings do not have the large scallop present at the tip of *ptc-Gal4 > uif* RNAi wings (Fig 3A), demonstrating that Mgl does not act with Uif in the growth-related function underlying this phenotype. Quantitative data, S8 Table

2 **The supernumerary chemosensory esos produced by loss of *uif* or *Notch* function are also produced by suppression of apoptosis**

**Fig 9:**
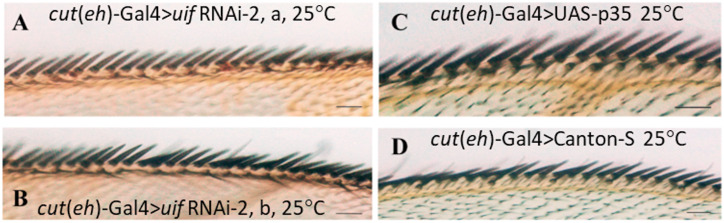
Generation of supernumerary chemosensory eso’s by *uif* knockdown phenocopies inhibition of apoptosis in the wing margin. Wing margins from *cut*(ue)-Gal4 crosses at 25^0^C. **A,** and **B.** Two stretches (a and b) of the anterior margin from a single *cut*(ue)-Gal4 > *uif* RNAi-2 wing, that had 31chemosensory bristles in total on the inner bristle row. **C.** *cut*(ue)-Gal4 > UAS-p35 wing margin. A stretch of 12 chemosensory sense organs is present in the inner bristle row. **D.** *cut*(ue)-Gal4 > Canton S control with five well-spaced chemosensory bristles in the inner margin row. Scale bars = 500 μm.

The wing margin undergoes two bursts of apoptosis during the early pupal stages and it has been shown [37] that suppressing this cell death with the baculovirus p35 protein produces additional chemosensory esos in the anterior wing margin. We compared the effects of p35 overexpression and *uif* knockdown on the margin esos using the *cut*(ue)-Gal4 driver at 25^0^C. p35 expression produced tight runs of chemosensory esos (Fig 9C) that appear identical to those formed by loss of *uif* activity (Fig 9A, B). A further shared property was the tendency for these runs to end after the junction of the L2 vein with the margin (Fig 9B, C). Quantitative data, S9 Table.

3 **Uif function in the microchaetes of the notum**

The claim that *uif* has a role in the differentiation of the notal esos is based on data for a small group of bristles on the notum edge. The main microchaete bristle arrays of the notum have not been studied [5]. Given that we found no role for Uif in wing margin eso differentiation, we investigated the effects of *uif* knockdown on the notal microchaetes, comparing any phenotypes detected to those produced by *Notch*, as in our other experiments. We used the *pannier* (*pnr*)-Gal4 driver for this investigation [38]. This Gal4 line expresses in a long anterior-posterior stripe through the ~eight central rows of microchaetes on the notum (Fig 1F).

**Fig 10:**
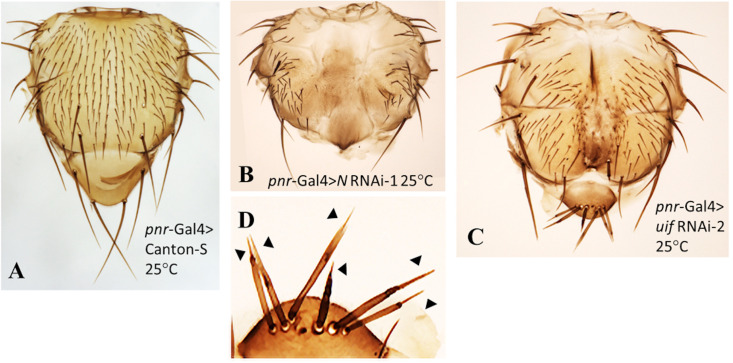
*uif* suppression produces growth and pigmentation defects in the microchaetes but does not alter cell fate. Thoraces from *pnr*-Gal4 crosses at 25^0^C. **A.** *pnr*-Gal4 > Canton-S. **B.** *pnr*-Gal4 > *Notch* RNAi. **C.** *pnr*-Gal4 > *uif* RNAi. **B.** Notch suppression produces total loss of microchaete bristles and notum areas without a cuticle (white patches). **C.** The length of the microchaete bristles in the mid-notum region is a function of their closeness to the center of the *pnr*-Gal4 stripe. At the actual center of the *pnr*-Gal4 stripe no bristles are present. Further examples of this phenomenon are shown in S2 Fig. **D.** Higher magnification image of the macrochaetes on the scutellum of the thorax in **C.** Arrowheads point to the shrivelled, pigment-free tips of these bristles. The trichomes on the notum cells also show a graded growth response to the gradient of *pnr*-Gal4 activity (S3 Fig).

At 25^0^C knockdown of *Notch* with the *pnr*-Gal4 driver produced a very strong phenotype (Fig 10A, B). Growth within the central notum was strongly inhibited, causing shortening of the entire thorax. The scutellum was not fully formed and lacked its characteristic four macrochaetes. The anterior and posterior ends of the *pnr* stripe region on all nota had large white patches of naked tissue without a cuticle, indicating a major role for *Notch* in cuticle formation. The central notum region was completely devoid of both microchaetes and macrochaetes and long-range disruption of *Notch* function (missing and malformed bristles outside the *pnr* stripe) was evident. The total absence of bristles in the *pnr* stripe suggests failure of microchaete development at a very early step in *Notch* regulation. Quantitative data, S10 Table.

Knockdown of *uif* with the *pnr*-Gal4 driver (Fig 10C) had clear similarities to the *pnr* Gal4 > *Notch* phenotype – patches of bare tissue with no cuticle were present at the same places on the thorax as in *Notch* knockdown, suggesting a function for *uif* in *Notch* regulation of cuticle synthesis. The scutellum was smaller than wild type and developed as a separate structure (Fig 10C). Effects on lateral bristles, distant from the central *pnr* stripe were also present, again indicating long-range effects of *the* knockdown.

This milder knockdown with *uif* RNAi resulted in the posterior region of the *pnr*-Gal4 stripe retaining microchaete bristles for analysis. The arrangement and shape of these bristles provided unexpected insight into how *uif* functions in their development. The bristles were such that **their length decreased as a function of their closeness to the center of the stripe of *pnr*-Gal4 expression**, **with the center of the stripe being devoid of bristles**. The simplest explanation for this behavior is that *pnr*-Gal4 produces a Gal4 gradient with the highest Gal4 levels at the center of its stripe and that the arrangement of the bristles reflects their response to an increasing gradient of loss of *uif* function. That response is loss of growth, which ultimately results in total loss of bristles at the stripe center. Two further examples of this pattern of microchaete growth for *pnr*-Gal4 > *uif* RNAi nota are shown in S2 Fig. No changes in cell fate were identified amongst these microchaete bristles. Further, the trichome hairs of the notal epidermal cells also showed a graded growth response to the *pnr*-Gal4 stripe in both *uif* and *Notch* knockdowns (S3 Fig), thus indicating growth defects in bristles in general. We also determined that the actual cells producing trichomes at the center of the *pnr*-stripe were smaller than those further out in the notum (S11 Table), suggesting that, ultimately, loss of trichome growth reflects failed growth of their parent cells.

On the scutellum, *uif* knockdown produced changes in the patterning and spacing of the four large bristles usually present. In several samples the bristles were mispositioned and all thoraces examined had additional or missing bristles (Fig 10D). These bristles showed growth and pigmentation defects. They were much shorter than usual and had slender, unpigmented needle-like tips (Fig 10D). Similar bristles were found on the thoraces themselves for both *uif* and *Notch* knockdown, in addition to occasional thick, misshaped bristles. Quantitative data, S10 Table

## Discussion

### Shared functions of *uif* and *Notch*

We have identified three functions for *uif* in the *Drosophila* wing. We have shown that *uif* plays roles in the growth of the wing blade and its margin resulting in failed wing expansion at eclosion in the absence of *uif* function. We have discovered a role for *uif in* regulation of pigment formation in the cuticle that results in ectopic pigment production in *uif* knockdown. Finally, and unexpectedly, we have found that loss of *uif* activity in the wing margin leads to over-production of chemosensory esos in the dorsal margin inner eso row, a phenotype previously shown to result from inhibition of apoptosis in the margin. All three of these roles for *uif* are shared with *Notch. Notch* is the dominant regulator of growth and differentiation in wing development and so it is perhaps not surprising that *uif* functions in pathways that are activated and controlled by *Notch*. Previous work has already demonstrated that Uif and Notch can dimerize *in vivo* and Uif-Notch complexes can be endocytosed into cells [5,16]. However, it should not be assumed that all of the shared roles presented here operate through this known interaction. Once activated, Notch becomes a transcription factor [39], and *uif* could be a downstream target gene for *Notch.* The multiple protein motifs in Uif’s extracellular domain may bind other external protein domains as steps in *Notch*-initiated pathways.

### No role for *uif* in cell fate functions of *Notch*

The C96-Gal4 driver allowed us to investigate a possible role for *uif* in *Notch* control of differentiation of the wing margin eso bristles. *Notch* knockdown with the C96-Gal4 driver produced a high level of pIIa cell transformations, mainly detected as double shafts on the stout mechanosensory bristles [9,40,41]. In addition, structures with three or four stout bristles, phenotypes not described previously, were present. The double shafts result from symmetrical division of the pIIa cell [41]. Greater suppression of *Notch* might result in symmetrical division of the eso SOP cell to produce two pIIa cells followed by symmetrical division of these two pIIa cells to yield the four trichogen cells needed for four-shaft structures. Interestingly, Hartenstein and Posakony [11] have provided evidence indicating that symmetrical division of pIIa cells is relatively common in wild type wings, affecting 25% of the stout bristle precursors. However, subsequently cells are swapped between neighboring esos to restore the normal arrangement of eso lineages. It seems possible that loss of a role for *Notch* during these two non-clonal events could play a role in the formation of the four-stout bristle structures.

*uif* knockdown at both 25^0^C and 29^0^C produced no real evidence of a role for *uif* in differentiation of the margin esos. Typically, the anterior wing margin houses 80–86 eso stout bristles [11]. The wings we had to analyze had incomplete anterior margins but, assuming 50% of these margins were present, the two examples of double stout bristles we found amongst 20 wings were two amongst ~800 stout bristles examined. Developmental processes are not immune to error. It seems likely that these structures simply reflect rare failures in eso differentiation. In particular, the transfer of cells between esos postulated by Hartenstein and Posakony stands out as a likely point for developmental error. On the notum, eso development is known to have a significant error rate. A 20% excess of cells is wrongly induced to begin microchaete differentiation and apoptosis is used to remove these cells [42].

The work claiming a role for Uif in differentiation of the notal esos [5] produced only a low level of eso cell fate transformations upon *uif* suppression, leading to the suggestion that further investigation was warranted [32]. Our examination of *uif f*unction in the microchaetes supports the conclusion that *uif* has no role in the differentiation of these cells. Fortunately, the *pnr*-Gal4 used in our studies appears to produce a gradient of loss of *uif* function across which we could examine the effects on the notal microchaetes. The only effect detected was progressive failure of bristle growth that ultimately led to total bristle loss in the center of the thorax. The trichome hairs of the notal cells also showed a *uif* RNAi-induced decrease in size according to their position in the *pnr*-Gal4 stripe (S3 Fig). Quantitating trichome density on the central notum established that the notal cells themselves are also smaller too (S11 Table). A similar situation appears to exist in the wing blade. The smaller blade cells produced by *uif* knockdown (S2 Table) appear to have smaller trichomes (Fig 3B). The effects of *uif* knockdown on hair/bristle growth may all be secondary to decreased cell growth.

C96-Gal4 > *Notch* RNAi wings also showed transformation of wing blade cells into vein tissue at the point of vein contact with the wing margin. This phenomenon represents a further example of *Notch’s* activity in cell differentiation in the wing. As for eso differentiation, this phenotype was not seen for C96-Gal4 > *uif* RNAi wings.

### Uif’s role in pigmentation and the cuticle

Loss of *uif* activity and to a lesser extent, *Notch* function with the *ptc*-Gal4 and C96-Gal4 drivers led to multiple forms of ectopic pigment production and distribution. Detailed understanding of how one of these defects (pigment puddles around the blade trichomes) is produced in *mgl* mutants [34] suggests a role for *uif* in endocytosis of the protein Yellow, or some other component of pigment generation, into the epithelial cells beneath the cuticle. Loss of *uif* function produced a stronger version of this “pigment pooling” phenomenon than loss of *mgl* function, but the fact that we were only able to analyze a single *mgl* RNAi construct weakens the significance of this observation. However, early in pupal life, levels of *uif* mRNA in the cuticle are 10 times higher than those of *mgl* mRNA [8] which argues for a greater role for *uif* in pigment regulation.

Two further findings of ours also suggest that Uif has additional roles in the cuticle. First, we found that loss of *uif* function leads to naked regions on the notum with no protective cuticle (Fig 10C). Second, wings with *uif* knockdown from C96-Gal4 > *uif* RNAi crosses at 29^0^C were generally quite brittle and easily broken, suggesting roles for *uif* in synthesis of unknown, structural elements in the cuticle.

A puzzling feature of the pigment defects we observed is that they were not limited to the expression patterns of the Gal4 drivers in question but rather showed a random distribution throughout the wings. Two prior studies suggest that this pigment distribution could be a consequence of events related to wing eclosion. If, during eclosion, the removal of the dead wing epithelial cells from the blade is incomplete, proper final fusion of the two cuticle layers is prevented, and random pigment patches appear throughout the wing [43]. This phenomenon has been termed “blemishing” [43]. A study by Hadjaje et al. [22] identifies a possible force for this pigment dispersal: at eclosion the wings are pressurized, and hemolymph floods the entire wing.

### Notch and Uif and apoptotic regulation of chemosensory eso production in the wing margin

Final structuring of the anterior wing margin requires two waves of apoptosis that are activated early in pupation [37]. Blocking this activity with baculovirus protein p35 results in over-production of chemosensory esos in the anterior wing margin [37]. We have shown that loss of *uif* function phenocopies this effect indicating that, in the wild type situation, *uif* is required for the apoptotic events. The p35 protein inhibits apoptosis by direct binding to the caspases [44]. Given the long extracellular domain of Uif and its known interactions with Notch [5,16], it seems likely that Uif’s engagement in these apoptotic events will entail a role in endocytosis. Endocytosis of surface receptors is a trigger for activating several apoptotic pathways including that of the *Drosophila* tumor necrosis factor Eiger [6,45]. It is now well established that components of the apoptotic pathways have functions in developmental processes [46,47]. In this case, *uif* appears to be part of a caspase-dependent developmental system that limits the number of chemosensory esos on the wing margin. The significance of this role is not immediately obvious. The functions of these chemosensors on wild type wings is not fully understood but roles in pheromone sensing [48] and grooming activities [49] induced by bacterial chemicals have been identified for some of them.

## Supporting information

S1 Fig Comparison of the effectiveness of two RNAi lines for *uif* and two for *Notch RNAi.* The phenotypes of the two *uif* RNAi’s used in these studies are very similar. This is also true for the two *Notch* RNAi’s used. **A.-D**. Examples demonstrating this point. **A.** *cut*(ue)-Gal4 > *uif* RNAi-1 wing. **B.** *cut*(ue)-Gal4 > *uif* RNAi-2. **C.** *cut*(ue)-Gal4 > *Notch* RNAi-1. **D**. *cut*(ue)-Gal4 > *Notch* RNAi-2.
(TIF)

S2 Fig Additional examples of the microchaete growth response to graded loss of *uif* function. **A, B.** Two further examples of the gradient of microchaete growth responses to graded *uif* suppression in the mid-region of the notum. In addition, aberrant bristles with altered growth and pigmentation are present throughout the notum (see black arrowheads). The circular object on thorax B. is an air bubble.
(TIF)

S3 Fig Trichomes show the same growth behavior as the microchaetes in relation to the *pnr*-Gal4 stripe. A. and B. show regions of the nota for the *pnr*-Gal4 > *uif* RNAi-2 and *pnr*-Gal4 > *Notch RNAi-2* genotypes, respectively. Anterior is up and the left-hand side of each image is the region of the *pnr*-stripe. Trichomes decrease is size (see arrows below images) as their position becomes closer to, or within, the *pnr-*Gal4 stripe. Note extremely small size of *Notch* trichomes.
(TIFF)

S1 Table *uif* implicated in wing blade growth and pigmentation. (XLSX)

S2 Table Cell density is higher in ptc-Gal4 > RNAi blade tissue than in control regions. (DOCX)

S3 Table Effects of *Notch* and *uif* knockdown on wing blade cell growth and pigmentation. (XLSX)

S4 Table *uif* knockdown in the wing margin results in mild Notch-like growth phenotypes. (XLSX)

S5 Table Further evidence for *Notch*-like margin growth function of *uif.* (XLSX)

S6 Table Defects in the wing margin specific to *Notch* knockdown. (XLSX)

S7 Table Shared defects in the wing margin from *Notch* and *uif* knockdown. (XLSX)

S8 Table Comparison of ectopic pigment and scallops on *mgl* and *uif* knockdown wings. (XLSX)

S9 Table Supernumerary chemosensory eso production by uif knockdown and p35 over expression. (XLSX)

S10 Table *uif* affects growth, not differentiation, of notal microchaete bristles. (XLSX)

S11 Table Increased trichome density on the notum indicates reduced notum cell size. (DOCX)
