# Supplementary material for: Functions of Uninflatable in the Drosophila melanogaster wing and notum
Source: PLoS One. 2026 May 15;21(5):e0344871. doi: 10.1371/journal.pone.0344871 (PMC13178913; doi:10.1371/journal.pone.0344871)
Supplement: S2 Table — (DOCX) [file pone.0344871.s005.docx]

| **Genotype** | **Temperature (°C)** | **Wings scored** | **Mean # trichomes / area** | **t-test p-value** |
| --- | --- | --- | --- | --- |
| *ptc*-Gal4>Canton-S | 25 | 22 | 56 |  |
| *ptc*-Gal4>*uif* RNAi-1 | 25 | 20 | 76 | 2.6 x 10^-10^ |

NIH Image J was used to count trichomes (each representing a single cell) in a defined area (8258 μm^2^) of the *ptc*-Gal4>*uif* RNAi stripe (see Figure 3) or an equivalent region of control tissue. Cell numbers were 50% higher in the *ptc*-Gal4 stripe indicating reduced cell growth upon suppression of *uif* activity.
