# Supplementary material for: Functions of Uninflatable in the Drosophila melanogaster wing and notum
Source: PLoS One. 2026 May 15;21(5):e0344871. doi: 10.1371/journal.pone.0344871 (PMC13178913; doi:10.1371/journal.pone.0344871)
Supplement: S11 Table — (DOCX) [file pone.0344871.s014.docx]

| **Genotype** | **Temperature (°C)** | **Nota scored** | **Mean # trichomes / area** | **t-test p-value** |
| --- | --- | --- | --- | --- |
| *pnr*-Gal4>Canton-S | 25 | 6 | 13 |  |
| *pnr*-Gal4>*Notch* RNAi | 25 | 4 | 22 | 0.038 |
| *pnr*-Gal4>*uif* RNAi | 25 | 6 | 19 | 0.018 |

NIH image J was used to compare trichome numbers within a defined area (20 μm^2^) of control, *uif* and *Notch* knockdown samples. The density of the smaller trichomes in the central notum regions of *uif* and *Notch* knockdown tissue indicate ~50% and ~70% increases respectively in cell density.
